# Proteostasis During Cerebral Ischemia

**DOI:** 10.3389/fnins.2019.00637

**Published:** 2019-06-19

**Authors:** Audrey M. Thiebaut, Elodie Hedou, Stefan J. Marciniak, Denis Vivien, Benoit D. Roussel

**Affiliations:** ^1^INSERM, INSERM UMR-S U1237, Physiopathology and Imaging of Neurological Disorders, University of Caen Normandy, Caen, France; ^2^Cambridge Institute for Medical Research, University of Cambridge, Cambridge, United Kingdom; ^3^Department of Medicine, Addenbrooke’s Hospital, University of Cambridge, Cambridge, United Kingdom; ^4^Department of Clinical Research, University of Caen Normandy, Caen, France

**Keywords:** stroke, autophagy, ER stress, proteostasis, mTOR

## Abstract

Cerebral ischemia is a complex pathology involving a cascade of cellular mechanisms, which deregulate proteostasis and lead to neuronal death. Proteostasis refers to the equilibrium between protein synthesis, folding, transport, and protein degradation. Within the brain proteostasis plays key roles in learning and memory by controlling protein synthesis and degradation. Two important pathways are implicated in the regulation of proteostasis: the unfolded protein response (UPR) and macroautophagy (called hereafter autophagy). Both are necessary for cell survival, however, their over-activation in duration or intensity can lead to cell death. Moreover, UPR and autophagy can activate and potentiate each other to worsen the issue of cerebral ischemia. A better understanding of autophagy and ER stress will allow the development of therapeutic strategies for stroke, both at the acute phase and during recovery. This review summarizes the latest therapeutic advances implicating ER stress or autophagy in cerebral ischemia. We argue that the processes governing proteostasis should be considered together in stroke, rather than focusing either on ER stress or autophagy in isolation.

## Introduction

Cerebral ischemia is a major cause of adult mortality and disability in developed countries. It is characterized by a reduction of cerebral blood flow, leading to a dramatic drop of energetic supply, altered cellular homeostasis and finally cell death (Doyle et al., 2008; Yepes et al., 2009). Several pathways leading to neuronal death are activated during ischemic events. Historically, glutamate receptor-mediated excitotoxicity, apoptosis and inflammation have been shown to be important parts of ischemia-mediated neuronal death (Dirnagl et al., 1999). More recently, ER stress via the UPR (Roussel et al., 2013) and autophagy (Liu et al., 2010) pathways, either at the acute phase or during recovery, has received much attention in the stroke research field.

To date, only two therapeutic approaches are available to promote reperfusion during ischemic stroke: the recent mechanical clot removal (endovascular thrombectomy), and thrombolysis by tPA (Thiebaut et al., 2018). Of note, tPA administration is often combined with endovascular thrombectomy, especially in patients with large vessel occlusion (Berkhemer et al., 2015). However, both treatments have their own limits: tPA has a narrow therapeutic window (4.5 h after stroke onset) (Hacke et al., 2008), presents a risk of hemorrhagic transformation and has a low recanalisation rate (National Institute of Neurological Disorders and Stroke rt-Pa Stroke Study Group, 1995; Thiebaut et al., 2018); while endovascular thrombectomy is restricted to large vessel occlusions (Berkhemer et al., 2015). Several strategies of neuroprotection have been tested in stroke, but no clinical trials have demonstrated any beneficial effect in patients (Young et al., 2007). To overcome this challenge, better stroke models and trial designs are required, and integration of multiple complex signaling pathways will be necessary to provide a complete understanding of stroke (Karsy et al., 2017).

Among such pathways, those maintaining proteostasis appear especially relevant as they encompass many responses, from changes in protein synthesis and folding to secretion and degradation. Moreover, altered proteostasis is implicated in both the acute and recovery phases of stroke (Wang and Yang, 2018). The UPR, which is the proteostasis control system affecting the ER, maintains the balance between protein synthesis, folding and degradation within the organelle (Roussel et al., 2013). On the other hand, protein or organelle degradation during stroke is mainly driven by autophagy (with ubiquitination and SUMOylation), and more particularly, macroautophagy (called hereafter autophagy) (Liu et al., 2010).

## Autophagy and Cerebral Ischemia

Autophagy is a protein degradation system highly conserved in all eukaryotes. Its name comes from Greek roots: “auto” which means “self” and “phagy” which means “eating.” This self-protecting catabolic process is required for restoring cellular homeostasis under nutrient starvation or metabolic stress by degrading or recycling long-lived or misfolded proteins and damaged organelles (Klionsky et al., 2016). Depending on the mode of cargo delivery, three types of autophagy have been described: microautophagy, CMA and macroautophagy.

**FIGURE 1:**
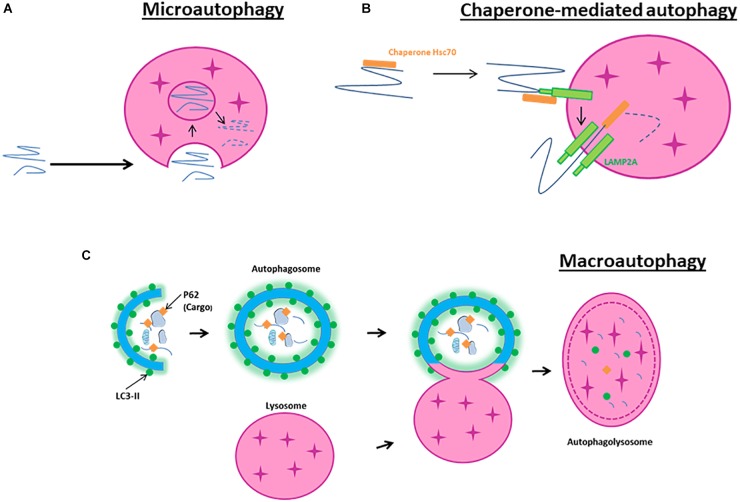
Schematic model of autophagy. **(A)** Macroautophagy is the result of the fusion of an autophagosome, a double membrane vacuole transporting cellular cargo that has been targeted for degradation, with a lysosome. **(B)** Microautophagy is a non-selective lysosomal degradative process that involves direct engulfment of cytoplasmic materials by the invagination of the lysosome. **(C)** Chaperone-mediated autophagy involves the direct translocation, through LAMP2A, of specific proteins containing a motif recognized by a chaperone named hsc70 (heat-shock cognate protein of 70 kDa).

Microautophagy is a non-selective lysosomal degradative process referring to as the direct engulfment of cytoplasmic materials by the lysosome. In microautophagy, the lysosomal membrane is randomly invaginated to trap portions of the cytosol (Figure 1A). Microautophagy is known to play several roles, such as the regulation of the ratio of lipid to protein at the lysosomal surface, the maintenance of organelle size, the membrane composition and lipid metabolism. It can occur simultaneously with macroautophagy or simply be a constitutive method to degrade long-lived proteins and membrane proteins (Iwata et al., 2005; Krick et al., 2009).

Chaperone-mediated autophagy is a selective lysosomal degradative process requiring the presence within the substrate protein of a specific amino acid motif recognized by the chaperone hsc70. All cargo recognized by hsc70 are delivered to lysosomes where they bind to the LAMP2A (Bandyopadhyay et al., 2008; Figure 1B). Then, they are translocated one-by-one into lysosomes assisted by a chaperone located in the lysosomal lumen. CMA contributes to protein quality control through selective removal of altered or damaged proteins (Zhang and Cuervo, 2008).

In contrast to microautophagy and CMA, macroautophagy involves sequestration of the cargo away from the lysosome. Macroautophagy requires the synthesis of an autophagosome, a double-layered membrane structure, to transport cellular cargo targeted for degradation. Autophagosomes then fuse with lysosomes to form autophagolysosomes resulting in the degradation of their contents (Figure 1C). Because macroautophagy is the most abundant and characterized form of autophagy, our review will primarily focus on its mechanisms and effects in cerebral ischemia.

Autophagy occurs at a basal level in all mammalian cells and is regulated by conditions such as starvation, oxidative stress or hypoxia, and can also be triggered by obesity, diabetes, inflammation and cancer. The induction of autophagy appears as a promising therapeutic strategy for neurodegenerative diseases with protein misfolding and aggregation (Alzheimer’s diseases, Parkinson’s disease, Tauopathies, Huntington’s disease, and various spinocerebellar ataxias) (Rubinsztein et al., 2012).

### Autophagy Mechanisms in Physiopathological Conditions

#### Autophagy Processes and Signaling Pathway

**FIGURE 2:**
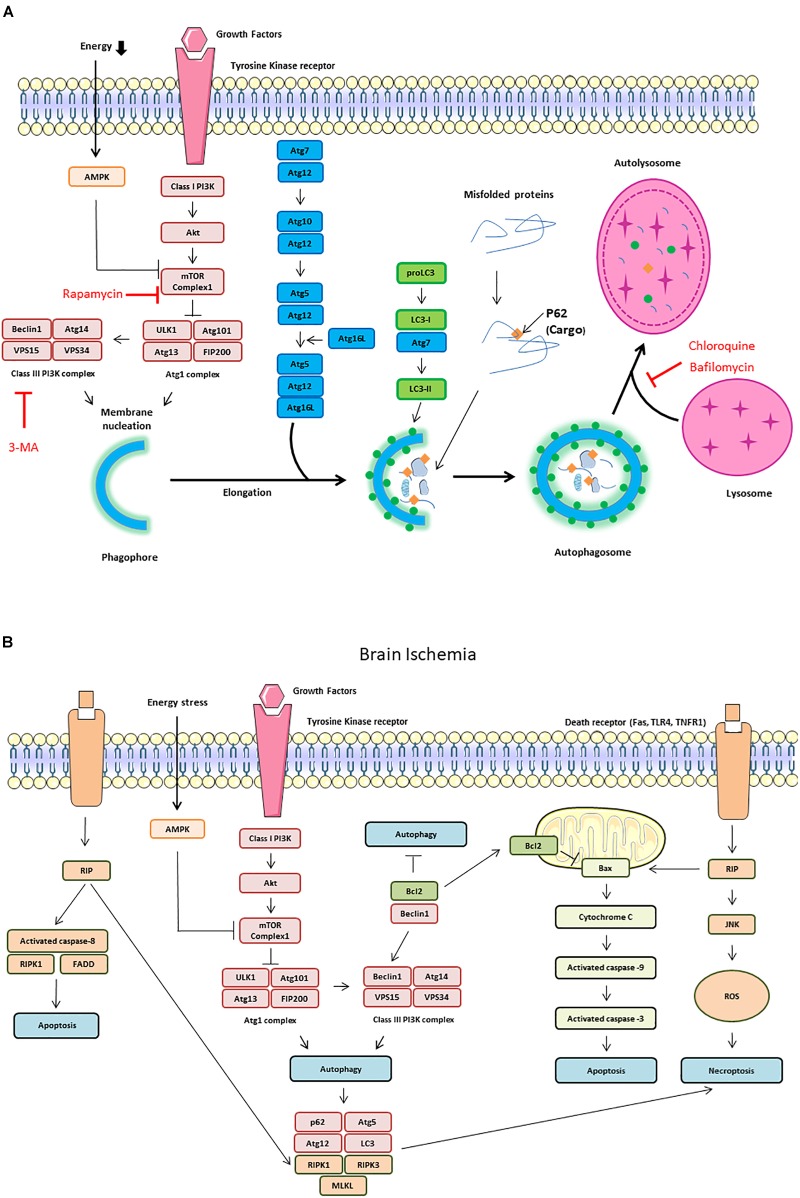
Autophagy signaling pathway. Autophagy is induced by an energetic stress, leading to AMPK activation; or by growth factors starvation, leading to the inhibition of the class I phosphatidylinositol 3-kinase (PI3K)/Akt/mammalian target of rapamycin (mTOR) pathway. Two complexes can be involved in autophagy induction: ULK1 complex composed of autophagy-regulated 101 (Agt1), Atg101, FIP200, and ULK1 and class III PI3K complex form by Beclin-1, vacuolar protein sorting 34 (VPS34) and vacuolar protein sorting 15 (VPS15). **(A)** Schematic illustration of autophagy initiation and autophagosome formation. Different Atg proteins including Atg5-Atg12-Atg16L and the microtubule-associated protein LC3-II are involved in double membrane elongation and in autophagosome-lysosome fusion. P62 binds to ubiquitinated proteins targeting them for degradation in the autolysosomes. Rapamycin can activate autophagy by inhibiting mTOR Complex 1. Both 3-MA (3-methyladenine), chloroquine and Bafilomycin are autophagy inhibitors by, respectively, inhibiting Class III PI3K complex and autophagosome/lysosome fusion. **(B)** Schematic illustration of cross talk and interaction among autophagy, apoptosis and necroptosis during brain ischemia. The dissociation of Beclin1 and Bcl-2 is also responsible of autophagy increase. The receptor interacting protein (RIP) Kinase 1 activation by death receptors, such as Fas, TLR4 and TNFR1, is responsible of Jun N-terminal kinase 1 (JNK1) pathway activation, ROS (reactive oxygen species) release and necroptosis. RIP1 interacts with FADD (Fas-Associated protein with Death Domain) and TRADD (Tumor necrosis factor receptor type 1-associated DEATH domain protein) to induce extrinsic apoptosis. RIP1 is also involved in intrinsic (mitochondrial) apoptosis through cytochrome C release which activates caspase-9 and capase-3.

The most common experimental approaches to induce autophagy are either nutrient starvation and or the use of rapamycin to inhibit the mTOR. Autophagy activates the ULK1 complex (ULK1, also known as Atg1 for intervention of autophagy-regulated 1), Atg13, and FIP200 (Noda and Ohsumi, 1998). The ULK1 complex also regulates the activation of a class III PI3K complex formed by Beclin-1, VPS34, and VPS15. When phosphorylated by ULK1, Beclin-1 promotes the generation of PI3P by VPS34, which upgrades autophagosomal membrane nucleation (Russell et al., 2013; Figure 2A). On the other hand, Beclin-1 can bind to the anti-apoptotic protein Bcl-2 leading to the inhibition of autophagy (Pattingre et al., 2005). In response to starvation, JNK1 phosphorylates Bcl-2 allowing its release from Beclin-1 and the induction of autophagy (Pattingre et al., 2009; Figure 2B).

In addition to these two complexes, other Atg proteins are hierarchically required for the biogenesis of autophagosomes. Atg5 and Atg12, co-operate with Atg7, to promote the formation of an Atg5-Atg12- Atg16L1 complex, and thus facilitate the cargo recruitment and autophagosomes membrane elongation. The microtubule-associated protein light chain 3 (LC3, also named Atg8) is cleaved by Atg4 to yield LC3-I, then, LC3-I is conjugated to PE by Atg7 and Atg3 to become LC3-II (Mizushima et al., 2011). p62, also called SQSTM1, binds to ubiquitinated proteins, targeting them for degradation into autolysosomes through its binding to LC3-II in the autophagosome (Myeku and Figueiredo-Pereira, 2011). LC3-II is essential for phagophore formation, with the participation of GABARAP/GATE-16 subfamily members in later stages of autophagosome formation, in particular for phagophore elongation, closure and fusion (Weidberg et al., 2010). Autophagosome can acquire membranes from multiple sources including the ER, the Golgi apparatus, the plasma membrane and the mitochondrial outer membrane. This process requires the involvement of the only transmembrane Atg protein, Atg9, which is present in single-membrane vesicles providing membranes for early autophagosome formation (Yamamoto et al., 2012). The fusion between the autophagosome and the lysosome requires the recruitment from the ER and mitochondria of the SNARE protein STX17. STX17 interacts with SNAP29 and VAMP8 to form a *trans*-SNARE complex and mediates autophagosome-lysosomes fusion (Itakura et al., 2012).

#### Autophagy Activation in Brain Ischemia

Nitatori et al. (1995) were the first to demonstrate, by electron microscopy, an increase in cathepsin B-immunopositive lysosomes, in the CA1 pyramidal neurons 3 days after an ischemic insult. Since then, the evidence for activation of autophagy has continued to accumulate. For example, beclin 1 is activated and associated to a LC3-II increase following neonatal hypoxia-ischemia (HI) (Carloni et al., 2008) and focal cerebral ischemia (Rami et al., 2008). In neonatal cerebral HI, in addition to the increase in autophagosomal formation (characterized by an increase of LC3-II), there is also an increase of lysosomal activity in damaged cortical and CA3 neurons, suggesting an increase in the autophagic flux (Ginet et al., 2009). These results are confirmed in a pMCAO. Moreover, immunofluorescence shows strong co-localisation of autophagosomal puncta (LC3-II) and lysosomes (cathepsin D and LAMP1) (Puyal et al., 2009). Furthermore, a study with 37 acute ischemic stroke patients revealed that CSF and serum levels of both Beclin-1 and LC3 are increased compared to control patients (Li et al., 2015), suggesting that autophagic cells die and release these markers in the circulation. Levels of Beclin1 and LC3 in CSF are positively correlated with infarct volumes and the severity of neurological deficits (NIHSS and Modified Rankin Scale) (Li et al., 2015). In addition, the expression of autophagic and apoptotic genes is increased in blood samples of ischemic stroke patients (Guo et al., 2017). However, Liu et al. (2010) reported that autophagy pathway is up-regulated after cerebral ischemia/reperfusion, but fails to operate correctly. They propose the idea that at least a part of the accumulation of protein aggregate-associated organelles seen following ischemia is due to the failure of autophagosomes to fuse with lysosomes (Liu et al., 2010).

After brain ischemia, autophagy-related signaling pathways are dramatically activated. In neonatal HI, Akt and CREB are activated in neurons and both phospho-Akt and phospho-CREB colocalized with Beclin-1. In this model, it has been shown that activation of autophagy activated by rapamycin was neuroprotective, and this was associated with the activation of Akt/CREB signaling (Carloni et al., 2010). During ischemia, a decrease in ATP concentration increases the AMP/ATP ratio and activates AMPK. Active AMPK leads to phosphorylation of TSC1/2 (tuberous sclerosis proteins 1 and 2) and inhibition of mTORC1 activity through Rheb. Several groups have reported the involvement of AMPK signaling pathway in the mediation of autophagy-related neuroprotection in brain ischemia (Wang et al., 2012; Gabryel et al., 2014; Jiang et al., 2014; Dai et al., 2017). Conversely, a study has shown that inhibition of AMPK activity inhibits autophagy and alleviates focal cerebral ischemia injury in mice by restoring mTORC1 activity (Fu et al., 2016). Autophagy may also be increased by the HIF-1α, a key transcription factor activated by the low oxygen conditions observed during cerebral ischemia (Xin et al., 2011; Yang et al., 2014; Zhang et al., 2018).

### Role of Autophagy in Cerebral Ischemia

While brain ischemia is known to induce autophagy, its protective or deleterious role remains unclear. Indeed, *in vitro* and *in vivo* studies report contradictory effects of autophagy induction during ischemia.

#### Neuronal Autophagy in the Ischemia Brain

Neuronal autophagy occurs early during cerebral ischemia, with autophagosomes and autolysosomes detectable just 1 h after pMCAo, and increasing up to 12 h thereafter (Wen et al., 2008). In line with this, Tian et al. (2010) showed, using GFP-LC3 transgenic mice, increased levels of autophagosomes in the ipsilateral hemisphere at 1, 3, and 6 days following tMCAo, with a peak at 1 day. The cells containing GFP-LC3-punctae were mostly neurons (Tian et al., 2010). Indeed, during cerebral ischemia, autophagy is predominantly a neuronal phenomenon (Carloni et al., 2008; Rami et al., 2008; Ginet et al., 2009; Puyal et al., 2009).

### Autophagy and Neuronal Death in Brain Ischemia

After cerebral ischemia, the number of GFP-LC3-punctae/TUNEL (terminal deoxynucleotidyl transferase-mediated dUTP-digoxigenin nick end labeling) double-positive cells increases in both the core and the peri-ischemic area (Tian et al., 2010). In addition, dying neurons displaying intense vacuolisation and numerous autophagosomes are detected 6 h after HI in neonatal rats, with a peak at 24 h. These dying neurons display some features of apoptosis such as chromatin condensation, cytoplasmic shrinkage, and relatively well-preserved organelles, suggesting that autophagy could precede apoptosis (Ginet et al., 2009). Uchiyama (2001) have shown that inhibiting autophagy protects neuron-like differentiated PC12 cells from apoptosis following serum deprivation, suggesting that autophagy is involved in neuronal cell death *in vitro*. This association between neuronal death and autophagy has led to the term “autophagic death” (Figure 2B). Beclin 1 has been reported to be involved in autophagic death. RNAi against Beclin-1 reduces neuronal autophagy and apoptosis in tMCAo model (Zheng et al., 2009). In addition, Beclin 1 knockdown inhibits autophagosomal formation and alleviates apoptotic neurodegeneration in the ipsilateral thalamus following focal ischemia (Xing et al., 2012).

Apoptosis is not the only mechanism involved in ischemia-induced cell death. In 2005, a study shown that an intracerebroventricular injection of necrostatin-1, an inhibitor of necroptosis (programmed necrosis), decreases the infarct volume in a mouse MCAo model (Degterev et al., 2005). A recent work demonstrates in a pMCAo model, that the RIP1K-regulated necroptosis contribute to ischemia-mediated neuronal and astrocytic cell death through the activation of autophagic pathways (Ni et al., 2018). Furthermore, necrostatin-1 decreases LC3-II level and autophagy (Ni et al., 2018). On the other hand, pre-treatment with 3-MA, an inhibitor of autophagy and PI3K, prevents neuronal necroptosis at 7 days of reperfusion in a severe model of global cerebral ischemia (Wang et al., 2011).

Despite controversial findings suggesting that ischemia induces autophagy only in the hippocampal CA1 region (Gao et al., 2015), it has been suggested that hippocampal CA1 and CA3 regions present a differential response to ischemia-induced autophagy, with CA1 neurons mostly undergoing apoptosis without autophagy, while CA3 neurons mostly undergo autophagic death (Ginet et al., 2009; Papadakis et al., 2013). The reasons for these subpopulation differences remain to be established.

### Deleterious Autophagy in Brain Ischemia

In both primary cultures of neurons and SH-SY5Y cells subjected to OGD, autophagic cell death occurs and can be reversed by 3-MA, a PI3Ks inhibitor (Shi et al., 2012). Bao et al. (2017) suggest that this effect is dependent of autophagy-induced AMPAR subunits GluR1, GluR2, and GluR3 upregulation during OGD, leading to an increase of cytoplasmic Ca^2+^ levels. In rats subjected to transient focal cerebral ischemia, intraventricular injection of 3-MA dramatically reduces the lesion volume (by 46%) whereas an inhibitor of apoptosis does not provide protection (Puyal et al., 2009). Several inhibitors of autophagy (3-MA, bafilomycin, and the cathepsin B inhibitor Z-FA-fmk) significantly reduce the increase of autophagosome and autolysosome, the downregulation of Bcl2, infarct volume, brain oedema and motor deficits in a rat model of pMCAO (Wen et al., 2008). Thus, it seems that decreasing autophagy both *in vitro* and *in vivo* during cerebral ischemia is beneficial (Cui et al., 2013; Jiang et al., 2017; Ryan et al., 2018).

Knocking down Beclin1 and Atg7 with siRNA reduce autophagy and excitotoxic cell death induced by both kainate and hypoxia in primary neurons (Ginet et al., 2014). Atg7 deficient mice show nearly complete protection from HI-induced caspase-3 activation and hippocampal pyramidal neuronal death (Koike et al., 2008). Furthermore selective neuronal deletion of Atg7 reduces autophagy and infarct volume by 42% in neonatal mice subjected to HI (Xie et al., 2016). Overexpression of microRNA-9a-5p (miR-9a-5p) decreases Atg5 protein level, leading to a decrease of infarct volume and neurological deficit in a rat model of MCAo (Wang et al., 2018).

### Protective Autophagy in Brain Ischemia

In Wang et al. (2012) have shown that 3-MA pre-treatment is deleterious in a rat model of MCAo. The inhibition of autophagy by 3-MA or wortmannin, both PI3Ks inhibitor, accelerates the progression toward necrotic cell death in neonatal HI model. Conversely, rapamycin, increases Beclin 1 expression and reduces necrotic cell death and brain damage (Carloni et al., 2008). Both ischemic preconditioning (IPC) and permanent focal ischemia induce autophagy activation by up-regulating LC3-II and Beclin-1. IPC treatment significantly reduces infarct volume, brain oedema and motor deficits, whereas 3-MA and bafilomycin suppress IPC-induced neuroprotection (Sheng et al., 2010). The neuroprotective action of rapamycin has been confirmed in several models of MCAo (Chauhan et al., 2011; Buckley et al., 2014). Finally, neuronal autophagy upon brain ischemia seems to be a part of pro-survival signaling pathway, that involves PI3K/Akt/TSC2 /mTOR/P70S6K signaling pathway (Wang et al., 2012; Papadakis et al., 2013) and Akt/CREB pathway (Carloni et al., 2010). IPC-induced autophagy is also neuroprotective and this effect has been suggested to be dependent of the amelioration of ER stress (Sheng et al., 2012). Inhibition of autophagy with Atg7 knock down increase ischemia-induced neuronal apoptosis in OGD and MCAo model. Mitochondrial clearance is reversed by 3-MA and Atg7 silencing, suggesting that mitophagy underlies the neuroprotection by autophagy (Zhang et al., 2013). Moreover, Atg7 silencing reverses the neuroprotection induced by an ER stress activator (Zhang et al., 2014). In fact both tunicamycin and thapsigargin protect against ischemic brain injury by activating mitochondrial autophagy during reperfusion. Interestingly, this effect is reversed by the inhibition of autophagy (Zhang et al., 2014). Knocking out arrestin-β1, which is upregulated after cerebral ischemia, protects neurons from OGD by impairing the interaction between Beclin-1 and PIK3 catalytic subunit type 3, thus decreasing autophagy (Wang et al., 2014). Beclin-1 seems central, as caveolin1, an integral membrane protein, is able to activate autophagy through its binding to Beclin-1/VPS34 complex: in Caveolin1 knock out mice, autophagy is impaired, leading to greater tMCAo-induced cerebral infarct (Nah et al., 2017).

#### Endothelial Autophagy in Brain Ischemia

In Engelhardt et al. (2015) have published a comparative study to characterize BBB-associated cells responses to HI. BMVECs exhibit greater responsiveness and sensitivity to OGD than astrocytes and pericytes. This is associated with the stabilization of HIF-1α, early disruption of cellular cytoskeleton and metabolism impairment, but unlike perivascular cells, BMVECs appear unable to induce autophagy. BMVECs are specialized endothelial cells, connected by tight junction and display intimate contact with supporting pericytes, astrocytes, microglia, and neurons in order to maintain the structural and functional integrity of the BBB. Brain ischemia and thrombolysis therapy with tPA induce BBB disruption, brain oedema and neuronal injury (Thiebaut et al., 2018). Several groups support the idea that autophagy induces endothelial cell injury and BBB disruption. Indeed focal ischemia, in NF-κB p50 knockout mice, induces autophagy like-injury in BMVECs together with a disruption of BBB integrity (Li et al., 2013). It appears that autophagy is beneficial for BMVECs and BBB integrity. In Li et al. (2014) shown that enhancing autophagy by rapamycin and lithium reverses OGD-induced collapsing of tight junction and decreases tMCAo-induced BBB leakage and brain oedema; while 3-MA intensifies BMVECs apoptosis. Supporting these results, the knockdown of Beclin-1 attenuates autophagy processes and reduces the viability of endothelial cells subjected to OGD, while treatment with rapamycin increases cell viability (Urbanek et al., 2014). Similarly, chloroquine-induced autophagy inhibition enhances BBB permeability during pMCAo in diabetic rats. By opposition to the results of Engelhart and collaborators, this group observed autophagy activation in BMVECs after ischemia (Fang et al., 2015).

#### Astrocytic Autophagy in Brain Ischemia

During brain ischemia, glial cells, especially astrocytes, play a crucial role. They are involved in water transport, ion homeostasis, glutamate uptake, cerebral blood flow regulation, cerebral inflammation, maintenance of the BBB integrity, modulation of neuroplasticity and the secretion of neurotrophic and neuroprotective factors (Nedergaard et al., 2003).

In Tian et al. (2010) have shown, in a mouse model of MCAo, that GFP-LC3-punctae positive cells were mostly neurons rather than astrocytes or microglial cells in the peri-ischemic area, suggesting that glial cells may be more resistant to brain ischemia-induced autophagy than neurons. Nonetheless, a few groups report that ischemia induces an increase of astrocytic autophagy (Qin et al., 2010; Zhang et al., 2010; Engelhardt et al., 2015). Autophagosomes start to appear in the cytoplasm of astrocytes within the ischemic cortex 1 h after pMCAo and in the cytoplasm of primary astrocytes 1 h after OGD, with a peak at 3 h (Qin et al., 2010). The number of lysosomes significantly increases 6 h after pMCAo and OGD. In addition, they observe an increase of LC3-II, Beclin-1, LAMP2 and lysosomal cathepsin B expression. 3-MA and bafilomycin decrease ischemia-induced autophagy, apoptosis and necrosis, suggesting that autophagic/lysosomal activation may contribute to ischemic injury of astrocytes (Qin et al., 2010). This is partially confirmed by the fact that RIP1K knockdown decreases autophagy, increases GFAP levels, and attenuates astrocytic necrotic cell death in the ischemic cortex (Ni et al., 2018). RIP1K-mediated necroptosis may play important roles in ischemia-induced neuronal and astrocytic cell death through the activation of autophagic-lysosomal pathway (Ni et al., 2018). In the same line, breviscapine provides neuroprotection in the penumbra by decreasing both astrocytic and neuronal autophagy induced by tMCAo (Pengyue et al., 2017).

In contrast, inhibition of AMPK, by siRNA targeting AMPKα1 or by a pharmacological inhibitor, attenuates autophagy and astrocyte viability following OGD. This work supports the idea that during ischemia AMPK is involved in the induction of protective autophagy in astrocytes (Gabryel et al., 2014). Furthermore, when the astrocytic autophagy is specifically enhanced, by using AAV-GFAP-ATG7, it leads to neuroprotection in astrocytes/neurons co-cultures exposed to OGD and improves neurological outcome following tMCAo (Liu et al., 2018).

It is important to mention that many inhibitors used in the autophagy field are not always selective. For example, 3-MA and wortmannin inhibit both class I and class III PI3K, and each class of PI3K has its own, and sometimes opposite, effect on autophagy (Petiot et al., 2000). Furthermore, the use of rapamycin to inhibit mTORC1 does not only affect autophagy, as mTORC1 is involved in cell growth, cell survival protein synthesis and ribosomes biogenesis (Boutouja et al., 2019). Rapamycin can also inhibit mTOR complex 2, a complex known to have pro-survival and pro-proliferation effect by increasing Akt and SGK (Boutouja et al., 2019). The use of these poorly selective autophagy inhibitors could explain some of the discrepancies observed in the literature.

## ER Stress and Cerebral Ischemia

Another important consequence of cerebral ischemia is ER stress. The ER is an important organelle for the folding of all secreted and membrane proteins. In stress conditions, such as ischemia, the ER cannot assume correctly its functions and triggers an adaptive program called the UPR, leading to two distinct actions: (i) an early inhibition of protein synthesis; (ii) a delayed upregulation of genes that promote protein folding and degradation (Marciniak and Ron, 2006).

In Def Webster and Ames (1965), it was first noted that neurons of the rabbit retinas show swelling of their ER when subjected to OGD. Although initially reversible, this change becomes irreversible when OGD lasts more than 30 min. Similar distension of the ER was subsequently reported in neurons in a rat model of MCAo reperfusion injury (Dietrich et al., 1989), implicating ER dysfunction in the complex process of ischemic neuronal damage.

**FIGURE 3:**
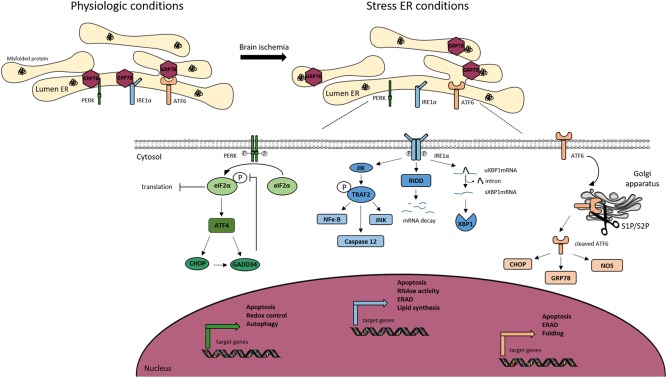
Endoplasmic reticulum (ER) stress signaling. Under physiological conditions, the Glucose Related Protein 78 (GRP78) binds to the three ER transmembrane sensors; protein kinase RNA-like ER kinase (PERK), inositol-requiring kinase 1α (IRE1α) and activating transcription factor 6 (ATF6), and maintains them inactive. Ischemic stroke induces ER stress, leading to the activation of the UPR. Under stress conditions, GRP78 dissociates from the ER sensors to bind to misfolded proteins, allowing the *trans*-autophosphorylation of PERK. P-PERK phosphorylates the eukaryotic translation initiation factor 2α (eIF2α), which decreases the global protein translation. Paradoxically, P-eIF2α allows the transcription of activating transcription factor 4 (ATF4), CCAT/enhancer binding protein homologous protein (CHOP). Both P-eIF2α and CHOP can activate the translation of growth arrest and DNA damage inducible gene/protein 34 (GADD34). The latter creates a feedback mechanism by dephosphorylating eiF2α. PERK is associated to apoptosis, autophagy and redox control. IRE1α dimerizes and *trans*-autophosphorylates, to splice the unspliced mRNA XBP1 (uXBP1) into spliced XBP1 (sXBP1). IRE1α activates c-jun-N-terminal-inhibiting kinase (JIK) and allows the recruitment of TNF receptor-associated factor 2 (TRAF2), leading to the activation of nuclear factor κ-B (NFκ-B), caspase 12 and c-jun-N-terminal kinase (JNK) pathway. IRE1α activates Regulated IRE1-Dependent Decay (RIDD) leading to the degradation of selected mRNAs. IRE1α is also involved in apoptosis, ER-associated degradation (ERAD) and lipid synthesis. The activation of ATF6 is dependent of its cleavage by S1P and S2P endopeptidases in the Golgi apparatus. Cleaved ATF6 induces the transcription of GRP78, nitric oxide synthase (NOS) and CHOP. Moreover, cleaved ATF6 controls ERAD, apoptosis and protein folding.

The threat of ER is detected in cells by three sensors that are present at the ER membrane, PERK, IRE1, and ATF, each mediating a separate branch of the UPR (Figure 3).

The PERK branch: the inhibition of protein synthesis is an early response to ER stress mediated by PERK (PKR-like endoplasmic reticulum eIF2α kinase) (Harding et al., 1999). Under physiological conditions, PERK is held inactive by its binding to the chaperone BiP (binding immunoglobulin protein, also known as Grp78). Upon stress, the kinase is activated by the release of BiP and phosphorylates its sole cytoplasmic target, eIF2α (Marciniak et al., 2006). This is a regulator of the initiation of protein synthesis. In teleological terms, a “goal” of the PERK branch activation is to prevent the further accumulation of misfolded proteins (Roussel et al., 2013). However, despite a global shutdown of protein translation due to the phosphorylation of eIF2α, some mRNAs are translated more efficiently (Harding et al., 2003). This is the case of ATF4, leading to a transcriptional program of stress-responsive genes including CHOP (C/EBP homologous protein) (Harding et al., 2003). The observation that Chop-/- animals and cells are resistant to ER stress, led some to suggest CHOP functions to promote ER stress-induced cell death. This is not fully supported, however, by analysis of the genes regulated by CHOP (Marciniak et al., 2004). Instead, CHOP appears to regulate a program that promotes the recovery of protein synthesis and secretion following the recovery of ER stress. For example, CHOP induces expression of GADD34 (protein phosphatase 1 regulatory subunit 15A), which is responsible for the dephosphorylation of eIF2α and thus the recovery of normal protein translation (Roussel et al., 2013). In this light, the ATF4-CHOP-GADD34 axis can be viewed as a negative feedback loop opposing the effects of PERK. It is therefore likely that the apparent “pro-death” role that has been attributed to CHOP is too simplistic. The recovery of translation is essential for normal functioning of the cell following mild ER stress, but perhaps in pathology, for example cerebral ischemia, there are benefits from disabling the normal rapid recovery of protein synthesis. This clearly has exciting therapeutic implications and many groups are currently developing approaches to antagonize the ATF4-CHOP-GADD34 axis for therapeutic benefit.

The ATF6 branch: like PERK, ATF6 is held inactive by the binding to BiP. Under stressed conditions, ATF6 is free to traffic to the Golgi apparatus where it is cleaved to release a soluble transcription factor for protein folding and degradation genes (Hillary and Fitzgerald, 2018).

The IRE1 branch: the activation of IRE1 is similar to that of ATF6 and PERK, but IRE1 is an endoribonuclease that triggers the splicing of the mRNA XBP1 (Calfon et al., 2002). The splicing induces a frameshift, allowing the active form of the protein. Proteins that fail to fold in the ER are eventually returned to the cytoplasm and targeted for degradation by the proteasome in process called ERAD. Many proteins of the ERAD machinery are induced by XBP1 (Yoshida et al., 2003). During intense ER stress, IRE1 also degrades many mRNAs located at the ER membrane through a mechanism called regulated IRE1-dependent decay (RIDD) (Hollien and Weissman, 2006). This helps further to reduce the load of new proteins entering the ER to be folded.

### UPR in Cerebral Ischemia

The ER is the main storage site for intracellular calcium, a central second messenger mediating diverse physiological processes in neurons, including secretion, synaptic plasticity and cell death (Zundorf and Reiser, 2011). Molecular chaperones that reside within the ER require high calcium concentrations to function efficiently (Michalak et al., 2002). This high calcium environment is maintained by the constant pumping of calcium from the cytosol into the ER by the SERCA (Doutheil et al., 1999b). Release of calcium back into the cytosol happens through the IP3R channel, which can be triggered by extracellular signals, or by the ryanodine receptor, activated by cytosolic calcium itself during CICR. Both lead to signaling but, if sustained, they deplete ER calcium stores and can induce chaperone dysfunction (Szydlowska and Tymianski, 2010). During cerebral ischemia, the influx of extracellular calcium via the NMDA receptor mediates glutamate-dependent neurotoxicity (Lopez-Atalaya et al., 2008). The rise in intracellular calcium triggers the release of ER calcium from ryanodine-sensitive stores, which is known to exacerbate CA1 hippocampal neuronal damage during global cerebral ischemia-reperfusion (Xing et al., 2004; Bull et al., 2008). Accordingly, inhibition of calcium release with the ryanodine receptor antagonist dantrolene decreases brain injury in animal models of both neonatal and adult HI (Wei and Perry, 1996; Gwak et al., 2008). While commonly attributed to the effects of cytosolic calcium, it is likely that effects on ER protein folding will also be important in these pathological conditions. Moreover, during cerebral ischemia, lack of ATP affects protein maturation (Kumar et al., 2003), and at the same time energy depletion leads to failure of the SERCA pumps (Doutheil et al., 1999b), contributing to the global ER calcium depletion and causing chaperone failure. Thus, the redistribution of calcium from ER to cytosol delivers a double blow to neurons, which exacerbates neurotoxicity (Paschen and Doutheil, 1999; Parsons et al., 1999).

The deregulation of ER-calcium homeostasis causing chaperone failure and activation of the UPR is readily apparent in many animal models of cerebral ischemia, including mice with BCAo (Kumar et al., 2001) or MCAo (Mengesdorf et al., 2002), rats with middle carotid artery occlusion with (Nakka et al., 2010) or without (Rissanen et al., 2006) reperfusion, cardiac arrest with reperfusion (Kumar et al., 2003) and a model of cortical spreading depression in rats (Schneeloch et al., 2004). This induction of ER stress during cerebral ischemia is responsible for the shutdown of protein translation seen during stroke (Burda et al., 1994; DeGracia et al., 1996; Hayashi et al., 2003; Kumar et al., 2003). It involves the activation of the stress-sensing PERK (Kumar et al., 2001; Li et al., 2005; Rissanen et al., 2006) that phosphorylates the translation initiation factor eIF2α, leading to the attenuation of new protein synthesis and disaggregation of polyribosomes (Althausen et al., 2001; Nakka et al., 2010).

Also, CHOP expression is elevated in several animal models of cerebral ischemia, including MCAo (Qi et al., 2004) and BCAo in mice (Tajiri et al., 2004; Osada et al., 2009), as well as MCAo and global ischemia in rats (Hayashi et al., 2005). Chop knockout (−/−) mice are more resistant to a BCAo type of ischemia than their wild-type littermates (Tajiri et al., 2004), and depletion of CHOP using RNA-interference in primary cultures of astrocytes partially prevents cell death in response to OGD (Benavides et al., 2005), suggesting that CHOP regulates a genetic program that is toxic in the context of cerebral ischemia. It has been shown that GADD34 is a target gene of CHOP responsible for some of the tissue damage seen in animal models of ER stress (Marciniak et al., 2004). This phosphatase is induced under conditions of cerebral ischemia (Doutheil et al., 1999a; Paschen et al., 2004; Li et al., 2005; Owen et al., 2005) and is detectable by immunostaining in the peri-infarct penumbra up to 24 h following the induction of stroke in rats (Imai et al., 2002; McCaig et al., 2005). As discussed already, it is therefore plausible that the toxicity attributed to CHOP is in fact the result of re-established protein translation mediated by GADD34 (Marciniak et al., 2004): it might be a mechanism implicated in reperfusion-mediated damage in cerebral ischemia. If this is the case, attenuation of protein translation should protect neurons from accumulating misfolded proteins. Consistent with this theory, salubrinal, a putative GADD34 inhibitor, has been shown to decrease ER stress and kainate-induced neurotoxicity *in vitro* (Sokka et al., 2007). However, the specificity of salubrinal has been questioned many times, since it was first demonstrated to work only *in vivo* (Boyce et al., 2005), and more recently its inhibitory effect has been shown to be really limited (Crespillo-Casado et al., 2017). IRE1, which splices the mRNA encoding the transcription factor XBP1, also shows activation during cerebral ischemia, for example in the cortex of mice after MCAo (Paschen et al., 2003; Morimoto et al., 2007) and BCAo (Tajiri et al., 2004). By contrast, ATF6 signaling remains a subject of debate: ATF6 expression was increased following MCAO in rats (Rissanen et al., 2006), but activation could not be demonstrated in an ischemia-reperfusion model by cardiac arrest in rats (Kumar et al., 2003).

## Crosstalk Between Autophagy and ER Stress

Autophagy and ER stress are important signaling pathways, but when dysregulated can lead to cell death, such like during cerebral ischemia. A well-balanced activation of these processes allow the maintenance or the recovery of proteostasis. However, their over-activation, by duration or intensity, tips the scale from a protective to a deleterious role (Roussel et al., 2013; Chen et al., 2014).

Although, as mentioned in the introduction, several interactions exist between ER stress and autophagy, in particular when misfolded proteins accumulate within the ER and activate PERK to induce autophagy (Kouroku et al., 2007). Moreover, downstream targets of PERK, like ATF4 and CHOP, induce the transcription of genes related to autophagy in response to both ER stress and amino acid starvation (B’Chir et al., 2013). During hypoxia, ATF4 facilitates autophagosome expansion through a binding to a cAMP response element-binding site within the *LC3b* promoter (Rzymski et al., 2010). Other sensors are also involved in autophagy induction, such as IRE1 by dissociating Beclin1 from its binding with Bcl2 (Wei et al., 2008). In addition, IRE1 also induces autophagy by activating AMPK in response to nitric oxide (Meares et al., 2011). XBP1, the mRNA processed by IRE1, is important for the negative feedback of ER stress-mediated autophagy, especially via its interaction with FOXO1 (Zhou et al., 2011). Interestingly, a genetic screen in *Drosophila* shows that both XBP1 loss of function and spliced-XBP1 overexpression lead to the induction of autophagy (Arsham and Neufeld, 2009). Finally, ATF6 has also been implicated in ER stress-mediated autophagy. By increasing the expression of DAPK1 (Gade et al., 2014), ATF6 promotes the dissociation of Beclin1 from Bcl2, leading to autophagy activation (Zalckvar et al., 2009).

In a mouse model of tMCAo, both ER stress and autophagy markers increase, suggesting a dual activation of ER stress and autophagy (Feng et al., 2017). It seems then important when studying proteostasis in cerebral ischemia, to pay attention to both phenomena and not only ER stress or autophagy.

Some studies also demonstrate an action of autophagy on ER stress. Indeed, the use of 3MA in a rat model of MCAo alters the ER stress response and leads to the stimulation of apoptosis and the enlargement of the cerebral infarct (Zhang et al., 2018). Similarly, Carloni et al. (2014) show a reduced ER stress activation and a better protection after neonatal HI when they increase autophagy with rapamycin. A possible explanation might be that CHOP induces death by inhibiting autophagy (Lei et al., 2017). This has been demonstrated in hepatocellular carcinoma cells, where ER stress inducer increases apoptosis and autophagy, while autophagy inhibitor promotes apoptosis and autophagy activator decreases ER stress-induced apoptosis (Lei et al., 2017). However, this hypothesis needs to be confirmed in neurons under ischemic/hypoxic challenge.

Finally, ER stress has been reported to cause the translocation of ER chaperones to the cell surface. BiP is the best described chaperone at the cell surface, and can act as a general co-receptor (Gonzalez-Gronow et al., 2009), with roles in autophagy, cancer and neuroprotection (Casas, 2017). Interestingly, cell-surface BiP can regulate autophagy by at least two mechanisms: by activating the PI3K/AKT pathway (Liu et al., 2013); and by associating to α2-macroglobulin receptor to activate mTOR signaling (Misra and Pizzo, 2012). Under OGD conditions, BiP is also at the neuronal surface (Louessard et al., 2017), and its binding to tPA results in a decrease in ER stress activation and neuroprotection (Louessard et al., 2017). In another model of hypoxia-induced apoptosis, the activation of neuronal-surface BiP by a synthetic peptide is also protective (Goldenberg-Cohen et al., 2012), reinforcing its role in neuroprotection during cerebral ischemia.

## Conclusion

Many neurological diseases, including cerebral ischemia, are associated with complete or partial activation of the UPR, perturbations in calcium homeostasis, autophagy dysfunctions, and finally cell death. Both an appreciation of the crucial role for ER homeostasis, the multiple forms that ER dysfunction can take, the regulation and the consequences of autophagy are now providing novel therapeutic strategies.

We propose a model in which proteostasis dysfunction spans a continuous spectrum from the UPR to autophagy, with probably a direct regulation of autophagy by the UPR as mentioned above. In cerebral ischemia, both pathways will be triggered to a greater or lesser degree, depending of stroke intensity, itself depending of the occlusion type and localisation. In each case, the relative contribution of each response and their interactions would need to be determined if rational approaches to therapy are to be developed.

Also, special attention should be given to studies using pharmacological approaches. As scientists, we all know that pharmacological treatments are really convenient, but we have to keep in mind that a “specific” drug has a lot of off targets actions. For example, it has been recently shown that GSK2606414, a PERK inhibitor, and KIRA6, an IRE1 inhibitor, are not only UPR specific (Mahameed et al., 2019). In fact they also inhibit KIT (also called CD117), a receptor tyrosine kinase involved in signal transduction pathways such as ERK and PI3K/AKT, upstream of mTOR and autophagy. In fact, many pharmacological activators and inhibitors are not as specific as they are supposed to be, and thus participate to discrepancies.

## Author Contributions

AT and EH did the research and wrote the autophagy and the ER stress part, respectively, and made the figures. SM has actively participated to the writing of the ER stress section and made useful comments on the whole manuscript. DV made corrections and comments on the manuscript. BR organized the review, wrote the introduction and the last section on the interactions between the autophagy and the ER stress, and made final corrections.

## Conflict of Interest Statement

The authors declare that the research was conducted in the absence of any commercial or financial relationships that could be construed as a potential conflict of interest.

## Abbreviations

3-MA: 3-methyladenine
AMP: adenosine monophosphate
AMPAR: α-amino-3-hydroxy-5-methyl-4-isoxazolepropionic acid receptor
AMPK: AMP-activated protein kinase
ATF4: activating transcription factor 4
ATF6: activating transcription factor 6
Atg: autophagy-regulated
Atg16L1: Atg16-like 1
ATP: adenosine triphosphate
Baf: bafilomycin
BBB: blood-brain barrier
BCAo: Bilateral cerebral artery occlusion
Bcl-2: B cell lymphoma 2
BiP: binding immunoglobulin protein
BMVECs: brain microvascular endothelial cells
CA: cornu ammonis
CHOP: CCAT/enhancer homologous protein
CICR: calcium-induced calcium release
CMA: chaperone-mediated autophagy
CREB: cAMP response element binding protein
CSF: cerebrospinal fluid
DAPK1: death-associated protein kinase 1
eIF2α: eukaryotic translation initiation factor 2α
ER: endoplasmic reticulum
ERAD: ER-associated protein degradation
FIP200: focal adhesion kinase family interacting protein of 200 kDa
FOXO1: forkhead box O1
GABARAP: -aminobutyric acid receptor-associated protein
GADD34: growth arrest and DNA damage inducible 34
GATE-16: Golgi-associated ATPase enhancer of 16 kDa
GFAP: glial fibrillary acidic protein
GFP: green fluorescence protein
Grp78: Glucose regulated protein 78
HI: hypoxia-ischemia
HIF-1α: hypoxia-inducible factor-1α
hsc70: heat-shock cognate protein of 70 kDa
IP3R: inositol trisphosphate receptor
IPC: ischemic preconditioning
IRE1: inositol-requiring enzyme 1
JNK1: Jun N-terminal kinase 1
LAMP2A: lysosomal-associated membrane protein 2A
LC3: light chain 3
miR-9a-5p: micro-RNA-9a-5p
mTOR: mammalian target of rapamycin
mTORC1: mammalian target of rapamycin complex 1
NF-κB: Nuclear factor-κB
NMDA: N-methyl-D-aspartate
OGD: oxygen and glucose deprivation
P70S6K: P70 ribosomal protein S6 kinase
PE: phosphatidylethanolamine
PERK: protein kinase R-like endoplasmic reticulum kinase
PI3K: phosphatidylinositol 3-kinase
PI3P: phosphatidylinositol 3-phosphate
pMCAO: permanent model of middle cerebral artery occlusion
Rheb: Ras homolog enriched in brain
RIP1K: receptor-interacting protein 1 kinase
SERCA: sarcoendoplasmic reticular calcium-ATPase
SGK: serum and glucocorticoid kinase
siRNA: small interfering RNA
SNAP29: synaptosomal-associated protein 29
SNARE: soluble N-ethylmaleimide-sensitive-factor attachment protein receptor
SQSTM1: sequestosome 1
STX17: syntaxin 17
tMCAo: transient MCAo
tPA: tissue-type plasminogen activator
TSC1/2: tuberous sclerosis complex 1 and 2
TSC2: tuberous sclerosis complex 2
ULK1: Unc-51-like kinase
UPR: unfolded protein response
VAMP8: vesicle-associated membrane protein 8
VPS15: vacuolar protein sorting 15
VPS34: vacuolar protein sorting 34
XBP1: X-box bonding protein 1.

## References

[B1] Althausen S. Mengesdorf T. Mies G. Olah L. Nairn A. C. Proud C. G. (2001). Changes in the phosphorylation of initiation factor eIF-2alpha, elongation factor eEF-2 and p70 S6 kinase after transient focal cerebral ischaemia in mice. *J. Neurochem.* 78 779–787. 10.1046/j.1471-4159.2001.00462.x 11520898

[B2] Arsham A. M. Neufeld T. P. (2009). A genetic screen in *Drosophila* reveals novel cytoprotective functions of the autophagy-lysosome pathway. *PLoS One* 29:e6068 10.1371/journal.pone.0006068 PMC2698153 19562034

[B3] Bandyopadhyay U. Kaushik S. Varticovski L. Cuervo A. M. (2008). The chaperone-mediated autophagy receptor organizes in dynamic protein complexes at the lysosomal membrane. *Mol. Cell. Biol.* 28 5747–5763. 10.1128/MCB.02070-07 18644871 PMC2546938

[B4] Bao L. Li R. H. Li M. Jin M. F. Li G. Han X. (2017). Autophagy-regulated AMPAR subunit upregulation in in vitro oxygen glucose deprivation/reoxygenation-induced hippocampal injury. *Brain Res.* 1 65–71. 10.1016/j.brainres.2017.05.019 28549968

[B5] B’Chir W. Maurin A. C. Carraro V. Averous J. Jousse C. Muranishi Y. (2013). The eIF2alpha/ATF4 pathway is essential for stress-induced autophagy gene expression. *Nucleic Acids Res.* 41 7683–7699. 10.1093/nar/gkt563 23804767 PMC3763548

[B6] Benavides A. Pastor D. Santos P. Tranque P. Calvo S. (2005). CHOP plays a pivotal role in the astrocyte death induced by oxygen and glucose deprivation. *Glia* 52 261–275. 10.1002/glia.20242 16001425

[B7] Berkhemer O. A. Fransen P. S. Beumer D. van den Berg L. A. Lingsma H. F. Yoo A. J. (2015). A randomized trial of intraarterial treatment for acute ischemic stroke. *N. Engl. J. Med.* 1 11–20. 10.1056/NEJMoa1411587 25517348

[B8] Boutouja F. Stiehm C. M. Platta H. W. (2019). mTOR: a cellular regulator interface in health and disease. *Cells* 2:8 10.3390/cells8010018 PMC6356367 30609721

[B9] Boyce M. Bryant K. F. Jousse C. Long K. Harding H. P. Scheuner D. (2005). A selective inhibitor of eIF2alpha dephosphorylation protects cells from ER stress. *Science* 11 935–939. 10.1126/science.1101902 15705855

[B10] Buckley K. M. Hess D. L. Sazonova I. Y. Periyasamy-Thandavan S. Barrett J. R. Kirks R. (2014). Rapamycin up-regulation of autophagy reduces infarct size and improves outcomes in both permanent MCAL, and embolic MCAO, murine models of stroke. *Exp.Transl. Stroke Med.* 6:8 10.1186/2040-7378-6-8 PMC4079187 24991402

[B11] Bull R. Finkelstein J. P. Galvez J. Sanchez G. Donoso P. Behrens M. I. (2008). Ischemia enhances activation by Ca2+ and redox modification of ryanodine receptor channels from rat brain cortex. *J. Neurosci.* 17 9463–9472. 10.1523/JNEUROSCI.2286-08.2008 PMC6671122 18799678

[B12] Burda J. Martin M. E. Garcia A. Alcazar A. Fando J. L. Salinas M. (1994). Phosphorylation of the alpha subunit of initiation factor 2 correlates with the inhibition of translation following transient cerebral ischaemia in the rat. *Biochem J.* 302(Pt 2), 335–338. 10.1042/bj3020335 8092984 PMC1137233

[B13] Calfon M. Zeng H. Urano F. Till J. H. Hubbard S. R. Harding H. P. (2002). IRE1 couples endoplasmic reticulum load to secretory capacity by processing the XBP-1 mRNA. *Nature* 3 92–96. 10.1038/415092a 11780124

[B14] Carloni S. Albertini M. C. Galluzzi L. Buonocore G. Proietti F. Balduini W. (2014). Increased autophagy reduces endoplasmic reticulum stress after neonatal hypoxia-ischemia: role of protein synthesis and autophagic pathways. *Exp. Neurol.* 255 103–112. 10.1016/j.expneurol.2014.03.002 24631374

[B15] Carloni S. Buonocore G. Balduini W. (2008). Protective role of autophagy in neonatal hypoxia-ischemia induced brain injury. *Neurobiol. Dis.* 32 329–339. 10.1016/j.nbd.2008.07.022 18760364

[B16] Carloni S. Girelli S. Scopa C. Buonocore G. Longini M. Balduini W. (2010). Activation of autophagy and Akt/CREB signaling play an equivalent role in the neuroprotective effect of rapamycin in neonatal hypoxia-ischemia. *Autophagy* 6 366–377. 10.4161/auto.6.3.11261 20168088

[B17] Casas C. (2017). GRP78 at the Centre of the Stage in Cancer and Neuroprotection. *Front. Neurosci.* 11:177 10.3389/fnins.2017.00177 PMC5380735 28424579

[B18] Chauhan A. Sharma U. Jagannathan N. R. Reeta K. H. Gupta Y. K. (2011). Rapamycin protects against middle cerebral artery occlusion induced focal cerebral ischemia in rats. *Behav. Brain Res.* 1 603–609. 10.1016/j.bbr.2011.08.035 21903138

[B19] Chen W. Sun Y. Liu K. Sun X. (2014). Autophagy: a double-edged sword for neuronal survival after cerebral ischemia. *Neural Regener. Res.* 15 1210–1216. 10.4103/1673-5374.135329 PMC4146291 25206784

[B20] Crespillo-Casado A. Chambers J. E. Fischer P. M. Marciniak S. J. Ron D. (2017). PPP1R15A-mediated dephosphorylation of eIF2alpha is unaffected by Sephin1 or Guanabenz. *eLife* 27:26109 10.7554/eLife.26109 PMC5429092 28447936

[B21] Cui D. R. Wang L. Jiang W. Qi A. H. Zhou Q. H. Zhang X. L. (2013). Propofol prevents cerebral ischemia-triggered autophagy activation and cell death in the rat hippocampus through the NF-kappaB/p53 signaling pathway. *Neuroscience* 29 117–132. 10.1016/j.neuroscience.2013.04.054 23644056

[B22] Dai S. H. Chen T. Li X. Yue K. Y. Luo P. Yang L. K. (2017). Sirt3 confers protection against neuronal ischemia by inducing autophagy: involvement of the AMPK-mTOR pathway. *Free Radical Biol. Med.* 108 345–353. 10.1016/j.freeradbiomed.2017.04.005 28396174

[B23] Def Webster H. Ames A. (1965). Reversible and irreversible changes in the fine structure of nervous tissue during oxygen and glucose deprivation. *J. Cell Biol.* 1 885–909. 10.1083/jcb.26.3.885 PMC2106794 19866686

[B24] DeGracia D. J. Neumar R. W. White B. C. Krause G. S. (1996). Global brain ischemia and reperfusion: modifications in eukaryotic initiation factors associated with inhibition of translation initiation. *J. Neurochem.* 67 2005–2012. 10.1046/j.1471-4159.1996.67052005.x 8863507

[B25] Degterev A. Huang Z. Boyce M. Li Y. Jagtap P. Mizushima N. (2005). Chemical inhibitor of nonapoptotic cell death with therapeutic potential for ischemic brain injury. *Nat. Chem. Biol.* 1 112–119. 10.1038/nchembio711 16408008

[B26] Dietrich W. D. Nakayama H. Watson B. D. Kanemitsu H. (1989). Morphological consequences of early reperfusion following thrombotic or mechanical occlusion of the rat middle cerebral artery. *Acta Neuropathol.* 78 605–614. 10.1007/bf00691287 2816302

[B27] Dirnagl U. Iaola C. Moskowitz M. A. (1999). Pathobiology of ischaemic stroke: an integrated view. *Trends Neurosci.* 22 391–397. 10.1016/s0166-2236(99)01401-0 10441299

[B28] Doutheil J. Althausen S. Gissel C. Paschen W. (1999a). Activation of MYD116 (gadd34) expression following transient forebrain ischemia of rat: implications for a role of disturbances of endoplasmic reticulum calcium homeostasis. *Brain Res. Mol. Brain Res.* 8 225–232. 10.1016/s0169-328x(98)00276-9 9878749

[B29] Doutheil J. Treiman M. Oschlies U. Paschen W. (1999b). Recovery of neuronal protein synthesis after irreversible inhibition of the endoplasmic reticulum calcium pump. *Cell Calcium* 25 419–428. 10.1054/ceca.1999.0042 10579053

[B30] Doyle K. P. Simon R. P. Stenzel-Poore M. P. (2008). Mechanisms of ischemic brain damage. *Neuropharmacology* 55 310–318. 10.1016/j.neuropharm.2008.01.005 18308346 PMC2603601

[B31] Engelhardt S. Huang S. F. Patkar S. Gassmann M. Ogunshola O. O. (2015). Differential responses of blood-brain barrier associated cells to hypoxia and ischemia: a comparative study. *Fluids Barriers CNS* 17:4 10.1186/2045-8118-12-4 PMC4429667 25879623

[B32] Fang L. Li X. Zhong Y. Yu J. Yu L. Dai H. (2015). Autophagy protects human brain microvascular endothelial cells against methylglyoxal-induced injuries, reproducible in a cerebral ischemic model in diabetic rats. *J. Neurochem.* 135 431–440. 10.1111/jnc.13277 26251121

[B33] Feng D. Wang B. Wang L. Abraham N. Tao K. Huang L. (2017). Pre-ischemia melatonin treatment alleviated acute neuronal injury after ischemic stroke by inhibiting endoplasmic reticulum stress-dependent autophagy via PERK and IRE1 signalings. *J. Pineal Res.* 62:e12395 10.1111/jpi.12395 28178380

[B34] Fu L. Huang L. Cao C. Yin Q. Liu J. (2016). Inhibition of AMP-activated protein kinase alleviates focal cerebral ischemia injury in mice: interference with mTOR and autophagy. *Brain Res.* 1 103–111. 10.1016/j.brainres.2016.08.035 27569585

[B35] Gabryel B. Kost A. Kasprowska D. Liber S. Machnik G. Wiaderkiewicz R. (2014). AMP-activated protein kinase is involved in induction of protective autophagy in astrocytes exposed to oxygen-glucose deprivation. *Cell Biol. Int.* 38 1086–1097. 10.1002/cbin.10299 24798185

[B36] Gade P. Manjegowda S. B. Nallar S. C. Maachani U. B. Cross A. S. Kalvakolanu D. V. (2014). Regulation of the death-associated protein kinase 1 expression and autophagy via ATF6 requires apoptosis signal-regulating kinase 1. *Mol. Cell. Biol.* 34 4033–4048. 10.1128/MCB.00397-14 25135476 PMC4386459

[B37] Gao C. Cai Y. Zhang X. Huang H. Wang J. Wang Y. (2015). Ischemic Preconditioning Mediates Neuroprotection against Ischemia in Mouse Hippocampal CA1 Neurons by Inducing Autophagy. *PLoS One* 10:e0137146 10.1371/journal.pone.0137146 PMC4556686 26325184

[B38] Ginet V. Puyal J. Clarke P. G. Truttmann A. C. (2009). Enhancement of autophagic flux after neonatal cerebral hypoxia-ischemia and its region-specific relationship to apoptotic mechanisms. *Am. J. Pathol.* 175 1962–1974. 10.2353/ajpath.2009.090463 19815706 PMC2774060

[B39] Ginet V. Spiehlmann A. Rummel C. Rudinskiy N. Grishchuk Y. Luthi-Carter R. (2014). Involvement of autophagy in hypoxic-excitotoxic neuronal death. *Autophagy* 10 846–860. 10.4161/auto.28264 24674959 PMC5119065

[B40] Goldenberg-Cohen N. Raiter A. Gaydar V. Dratviman-Storobinsky O. Goldstein T. Weizman A. (2012). Peptide-binding GRP78 protects neurons from hypoxia-induced apoptosis. *Apoptosis* 17 278–288. 10.1007/s10495-011-0678-x 22120956

[B41] Gonzalez-Gronow M. Selim M. A. Papalas J. Pizzo S. V. (2009). GRP78: a multifunctional receptor on the cell surface. *Antioxid. Redox Signal.* 11 2299–2306. 10.1089/ARS.2009.2568 19331544

[B42] Guo Y. Ma Y. Zhang Y. Zhou L. Huang S. Wen Y. (2017). Autophagy-related gene microarray and bioinformatics analysis for ischemic stroke detection. *Biochem. Biophys. Res. Commun.* 15 48–55. 10.1016/j.bbrc.2017.05.099 28528975

[B43] Gwak M. Park P. Kim K. Lim K. Jeong S. Baek C. (2008). The effects of dantrolene on hypoxic-ischemic injury in the neonatal rat brain. *Anesth. Analog.* 106 227–233. 10.1213/01.ane.0000287663.81050.38 18165582

[B44] Hacke W. Kaste M. Bluhmki E. Brozman M. Davalos A. Guidetti D. (2008). Thrombolysis with alteplase 3 to 4.5 hours after acute ischemic stroke. *N. Engl. J. Med.* 25 1317–1329. 10.1056/NEJMoa0804656 18815396

[B45] Harding H. P. Zhang Y. Ron D. (1999). Protein translation and folding are coupled by an endoplasmic-reticulum-resident kinase. *Nature* 21 271–274. 10.1038/16729 9930704

[B46] Harding H. P. Zhang Y. Zeng H. Novoa I. Lu P. D. Calfon M. (2003). An integrated stress response regulates amino acid metabolism and resistance to oxidative stress. *Mol. Cell* 11 619–633. 10.1016/s1097-2765(03)00105-9 12667446

[B47] Hayashi T. Saito A. Okuno S. Ferrand-Drake M. Chan P. H. (2003). Induction of GRP78 by ischemic preconditioning reduces endoplasmic reticulum stress and prevents delayed neuronal cell death. *J. Cereb. Blood Flow Metab.* 23 949–961. 10.1097/01.wcb.0000077641.41248.ea 12902839

[B48] Hayashi T. Saito A. Okuno S. Ferrand-Drake M. Dodd R. L. Chan P. H. (2005). Damage to the endoplasmic reticulum and activation of apoptotic machinery by oxidative stress in ischemic neurons. *J. Cereb. Blood Flow Metab.* 25 41–53. 10.1038/sj.jcbfm.9600005 15678111

[B49] Hillary R. F. Fitzgerald U. (2018). A lifetime of stress: ATF6 in development and homeostasis. *J. Biomed. Sci.* 25:48 10.1186/s12929-018-0453-1 PMC5968583 29801500

[B50] Hollien J. Weissman J. S. (2006). ay of endoplasmic reticulum-localized mRNAs during the unfolded protein response. *Science* 7 104–107. 10.1126/science.1129631 16825573

[B51] Imai H. Harland J. McCulloch J. Graham D. I. Brown S. M. Macrae I. M. (2002). Specific expression of the cell cycle regulation proteins, GADD34 and PCNA, in the peri-infarct zone after focal cerebral ischaemia in the rat. *Eur. J. Neurosci.* 15 1929–1936. 10.1046/j.1460-9568.2002.02025.x 12099899

[B52] Itakura E. Kishi-Itakura C. Mizushima N. (2012). The hairpin-type tail-anchored SNARE syntaxin 17 targets to autophagosomes for fusion with endosomes/lysosomes. *Cell* 7 1256–1269. 10.1016/j.cell.2012.11.001 23217709

[B53] Iwata A. Christianson J. C. Bucci M. Ellerby L. M. Nukina N. Forno L. S. (2005). Increased susceptibility of cytoplasmic over nuclear polyglutamine aggregates to autophagic degradation. *Proc. Natl. Acad. Sci. U.S.A.* 13 13135–13140. 10.1073/pnas.0505801102 PMC1201602 16141322

[B54] Jiang T. Yu J. T. Zhu X. C. Wang H. F. Tan M. S. Cao L. (2014). Acute metformin preconditioning confers neuroprotection against focal cerebral ischaemia by pre-activation of AMPK-dependent autophagy. *Br. J. Pharmacol.* 171 3146–3157. 10.1111/bph.12655 24611741 PMC4080970

[B55] Jiang W. W. Huang B. S. Han Y. Deng L. H. Wu L. X. (2017). Sodium hydrosulfide attenuates cerebral ischemia/reperfusion injury by suppressing overactivated autophagy in rats. *FEBS Open Biol.* 7 1686–1695. 10.1002/2211-5463.12301 PMC5666398 29123977

[B56] Karsy M. Brock A. Guan J. Taussky P. Kalani M. Y. Park M. S. (2017). Neuroprotective strategies and the underlying molecular basis of cerebrovascular stroke. *Neurosurg. Focus* 42:E3 10.3171/2017.1.FOCUS16522 28366066

[B57] Klionsky D. J. Abdelmohsen K. Abe A. Abedin M. J. Abeliovich H. Acevedo Arozena A. (2016). Guidelines for the use and interpretation of assays for monitoring autophagy (3rd edition). *Autophagy* 12 1–222. 26799652 10.1080/15548627.2015.1100356 PMC4835977

[B58] Koike M. Shibata M. Tadakoshi M. Gotoh K. Komatsu M. Waguri S. (2008). Inhibition of autophagy prevents hippocampal pyramidal neuron death after hypoxic-ischemic injury. *Am. J. Pathol.* 172 454–469. 10.2353/ajpath.2008.070876 18187572 PMC2312361

[B59] Kouroku Y. Fujita E. Tanida I. Ueno T. Isoai A. Kumagai H. (2007). ER stress (PERK/eIF2alpha phosphorylation) mediates the polyglutamine-induced LC3 conversion, an essential step for autophagy formation. *Cell Death Differ.* 14 230–239. 10.1038/sj.cdd.4401984 16794605

[B60] Krick R. Muhe Y. Prick T. Bredschneider M. Bremer S. Wenzel D. (2009). Piecemeal microautophagy of the nucleus: genetic and morphological traits. *Autophagy* 5 270–272. 10.4161/auto.5.2.7639 19182523

[B61] Kumar R. Azam S. Sullivan J. M. Owen C. Cavener D. R. Zhang P. (2001). Brain ischemia and reperfusion activates the eukaryotic initiation factor 2alpha kinase. *PERK. J. Neurochem.* 77 1418–1421. 10.1046/j.1471-4159.2001.00387.x 11389192

[B62] Kumar R. Krause G. S. Yoshida H. Mori K. DeGracia D. J. (2003). Dysfunction of the unfolded protein response during global brain ischemia and reperfusion. *J. Cereb. Blood Flow Metab.* 23 462–471. 10.1097/01.wcb.0000056064.25434.ca 12679723

[B63] Lei Y. Wang S. Ren B. Wang J. Chen J. Lu J. (2017). CHOP favors endoplasmic reticulum stress-induced apoptosis in hepatocellular carcinoma cells via inhibition of autophagy. *PLoS One* 12:e0183680 10.1371/journal.pone.0183680 PMC5571976 28841673

[B64] Li F. Hayashi T. Jin G. Deguchi K. Nagotani S. Nagano I. (2005). The protective effect of dantrolene on ischemic neuronal cell death is associated with reduced expression of endoplasmic reticulum stress markers. *Brain Res.* 28 59–68. 10.1016/j.brainres.2005.04.058 15921666

[B65] Li H. Gao A. Feng D. Wang Y. Zhang L. Cui Y. (2014). Evaluation of the protective potential of brain microvascular endothelial cell autophagy on blood-brain barrier integrity during experimental cerebral ischemia-reperfusion injury. *Transl. Stroke Res.* 5 618–626. 10.1007/s12975-014-0354-x 25070048

[B66] Li H. Qiu S. Li X. Li M. Peng Y. (2015). Autophagy biomarkers in CSF correlates with infarct size, clinical severity and neurological outcome in AIS patients. *J. Transl. Med.* 14:359 10.1186/s12967-015-0726-3 PMC4650838 26576535

[B67] Li W. L. Yu S. P. Chen D. Yu S. S. Jiang Y. J. Genetta T. (2013). The regulatory role of NF-kappaB in autophagy-like cell death after focal cerebral ischemia in mice. *Neuroscience* 6 16–30. 10.1016/j.neuroscience.2013.03.045 PMC3916093 23558089

[B68] Liu C. Gao Y. Barrett J. Hu B. (2010). Autophagy and protein aggregation after brain ischemia. *J. Neurochem.* 115 68–78. 10.1111/j.1471-4159.2010.06905.x 20633207 PMC3518272

[B69] Liu R. Li X. Gao W. Zhou Y. Wey S. Mitra S. K. (2013). Monoclonal antibody against cell surface GRP78 as a novel agent in suppressing PI3K/AKT signaling, tumor growth, and metastasis. *Clin. Cancer Res.* 15 6802–6811. 10.1158/1078-0432.CCR-13-1106 PMC4151476 24048331

[B70] Liu X. Tian F. Wang S. Wang F. Xiong L. (2018). Astrocyte autophagy flux protects neurons against oxygen-glucose deprivation and ischemic/ reperfusion injury. *Rejuvenation Res.* 21 405–415. 10.1089/rej.2017.1999 29125039

[B71] Lopez-Atalaya J. P. Roussel B. D. Levrat D. Parcq J. Nicole O. Hommet Y. (2008). Toward safer thrombolytic agents in stroke: molecular requirements for NMDA receptor-mediated neurotoxicity. *J. Cereb. Blood Flow Metab.* 28 1212–1221. 10.1038/jcbfm.2008.14 18334994

[B72] Louessard M. Bardou I. Lemarchand E. Thiebaut A. M. Parcq J. Leprince J. (2017). Activation of cell surface GRP78 reases endoplasmic reticulum stress and neuronal death. *Cell Death Differ.* 24 1518–1529. 10.1038/cdd.2017.35 28644439 PMC5563983

[B73] Mahameed M. Wilhelm T. Darawshi O. Obiedat A. Tommy W. S. Chintha C. (2019). The unfolded protein response modulators GSK2606414 and KIRA6 are potent KIT inhibitors. *Cell Death Dis.* 1:300 10.1038/s41419-019-1523-3 PMC6443726 30931942

[B74] Marciniak S. J. Garcia-Bonilla L. Hu J. Harding H. P. Ron D. (2006). Activation-dependent substrate recruitment by the eukaryotic translation initiation factor 2 kinase PERK. *J. Cell Biol.* 16 201–209. 10.1083/jcb.200508099 PMC2063550 16418533

[B75] Marciniak S. J. Ron D. (2006). Endoplasmic reticulum stress signaling in disease. *Physiol. Rev.* 86 1133–1149. 10.1152/physrev.00015.2006 17015486

[B76] Marciniak S. J. Yun C. Y. Oyadomari S. Novoa I. Zhang Y. Greis R. (2004). CHOP induces death by promoting protein synthesis and oxidation in the stressed endoplasmic reticulum. *Genes Dev.* 15 3066–3077. 10.1101/gad.1250704 PMC535917 15601821

[B77] McCaig D. Imai H. Gallagher L. Graham D. I. Harland J. Moira Brown S. (2005). Evolution of GADD34 expression after focal cerebral ischaemia. *Brain Res.* 9 51–61. 10.1016/j.brainres.2004.11.058 15713259

[B78] Meares G. P. Hughes K. J. Naatz A. Papa F. R. Urano F. Hansen P. A. (2011). IRE1-dependent activation of AMPK in response to nitric oxide. *Mol. Cell. Biol.* 31 4286–4297. 10.1128/MCB.05668-11 21896783 PMC3209336

[B79] Mengesdorf T. Proud C. G. Mies G. Paschen W. (2002). Mechanisms underlying suppression of protein synthesis induced by transient focal cerebral ischemia in mouse brain. *Exp. Neurol.* 177 538–546. 10.1006/exnr.2002.8002 12429199

[B80] Michalak M. Robert Parker J. M. Opas M. (2002). Ca2+ signaling and calcium binding chaperones of the endoplasmic reticulum. *Cell Calcium* 32 269–278. 10.1016/s0143416002001884 12543089

[B81] Misra U. K. Pizzo S. V. (2012). Receptor-recognized alpha(2)-macroglobulin binds to cell surface-associated GRP78 and activates mTORC1 and mTORC2 signaling in prostate cancer cells. *PLoS One* 7:e51735 10.1371/journal.pone.0051735 PMC3522726 23272152

[B82] Mizushima N. Yoshimori T. Ohsumi Y. (2011). The role of Atg proteins in autophagosome formation. *Annu. Rev. Cell Dev. Biol.* 27 107–132. 10.1146/annurev-cellbio-092910-154005 21801009

[B83] Morimoto N. Oida Y. Shimazawa M. Miura M. Kudo T. Imaizumi K. (2007). Involvement of endoplasmic reticulum stress after middle cerebral artery occlusion in mice. *Neuroscience* 29 957–967. 10.1016/j.neuroscience.2007.04.017 17590517

[B84] Myeku N. Figueiredo-Pereira M. E. (2011). Dynamics of the degradation of ubiquitinated proteins by proteasomes and autophagy: association with sequestosome 1/p62. *J. Biol. Chem.* 24 22426–22440. 10.1074/jbc.M110.149252 PMC3121389 21536669

[B85] Nah J. Yoo S. M. Jung S. Jeong E. I. Park M. Kaang B. K. (2017). Phosphorylated CAV1 activates autophagy through an interaction with BECN1 under oxidative stress. *Cell Death Dis.* 25:e2822 10.1038/cddis.2017.71 PMC5520747 28542134

[B86] Nakka V. P. Gusain A. Raghubir R. (2010). Endoplasmic reticulum stress plays critical role in brain damage after cerebral ischemia/reperfusion in rats. *Neurotox Res.* 17 189–202. 10.1007/s12640-009-9110-5 19763736

[B87] National Institute of Neurological Disorders and Stroke rt-Pa Stroke Study Group (1995). Tissue plasminogen activator for acute ischemic stroke. *N. Engl. J. Med.* 14 1581–1587. 10.1056/NEJM199512143332401 7477192

[B88] Nedergaard M. Ransom B. Goldman S. A. (2003). New roles for astrocytes: redefining the functional architecture of the brain. *Trends Neurosci.* 26 523–530. 10.1016/j.tins.2003.08.008 14522144

[B89] Ni Y. Gu W. W. Liu Z. H. Zhu Y. M. Rong J. G. Kent T. A. (2018). RIP1K Contributes to Neuronal and Astrocytic Cell Death in Ischemic Stroke via Activating Autophagic-lysosomal Pathway. *Neuroscience* 10 60–74. 10.1016/j.neuroscience.2017.10.038 29102662

[B90] Nitatori T. Sato N. Waguri S. Karasawa Y. Araki H. Shibanai K. (1995). Delayed neuronal death in the CA1 pyramidal cell layer of the gerbil hippocampus following transient ischemia is apoptosis. *J. Neurosci.* 15 1001–1011. 10.1523/jneurosci.15-02-01001.1995 7869078 PMC6577848

[B91] Noda T. Ohsumi Y. (1998). Tor, a phosphatidylinositol kinase homologue, controls autophagy in yeast. *J. Biol. Chem.* 13 3963–3966. 10.1074/jbc.273.7.3963 9461583

[B92] Osada N. Kosuge Y. Kihara T. Ishige K. Ito Y. (2009). Apolipoprotein E-deficient mice are more vulnerable to ER stress after transient forebrain ischemia. *Neurochem. Int.* 54 403–409. 10.1016/j.neuint.2009.01.010 19428781

[B93] Owen C. R. Kumar R. Zhang P. McGrath B. C. Cavener D. R. Krause G. S. (2005). PERK is responsible for the increased phosphorylation of eIF2alpha and the severe inhibition of protein synthesis after transient global brain ischemia. *J. Neurochem.* 94 1235–1242. 10.1111/j.1471-4159.2005.03276.x 16000157

[B94] Papadakis M. Hadley G. Xilouri M. Hoyte L. C. Nagel S. McMenamin M. M. (2013). Tsc1 (hamartin) confers neuroprotection against ischemia by inducing autophagy. *Nat. Med.* 19 351–357. 10.1038/nm.3097 23435171 PMC3744134

[B95] Parsons J. T. Churn S. B. DeLorenzo R. J. (1999). Global ischemia-induced inhibition of the coupling ratio of calcium uptake and ATP hydrolysis by rat whole brain microsomal Mg(2+)/Ca(2+) ATPase. *Brain Res.* 10 32–41. 10.1016/s0006-8993(99)01504-8 10407091

[B96] Paschen W. Aufenberg C. Hotop S. Mengesdorf T. (2003). Transient cerebral ischemia activates processing of xbp1 messenger RNA indicative of endoplasmic reticulum stress. *J. Cereb. Blood Flow Metab.* 23 449–461. 10.1097/00004647-200304000-00009 12679722

[B97] Paschen W. Doutheil J. (1999). Disturbances of the functioning of endoplasmic reticulum: a key mechanism underlying neuronal cell injury? *J. Cereb. Blood Flow Metab.* 19 1–18. 10.1097/00004647-199901000-00001 9886350

[B98] Paschen W. Hayashi T. Saito A. Chan P. H. (2004). GADD34 protein levels increase after transient ischemia in the cortex but not in the CA1 subfield: implications for post-ischemic recovery of protein synthesis in ischemia-resistant cells. *J. Neurochem.* 90 694–701. 10.1111/j.1471-4159.2004.02555.x 15255948

[B99] Pattingre S. Bauvy C. Carpentier S. Levade T. Levine B. Codogno P. (2009). Role of JNK1-dependent Bcl-2 phosphorylation in ceramide-induced macroautophagy. *J. Biol. Chem.* 30 2719–2728. 10.1074/jbc.M805920200 PMC2631952 19029119

[B100] Pattingre S. Tassa A. Qu X. Garuti R. Liang X. H. Mizushima N. (2005). Bcl-2 antiapoptotic proteins inhibit Beclin 1-dependent autophagy. *Cell* 23 927–939. 10.1016/j.cell.2005.07.002 16179260

[B101] Pengyue Z. Tao G. Hongyun H. Liqiang Y. Yihao D. (2017). Breviscapine confers a neuroprotective efficacy against transient focal cerebral ischemia by attenuating neuronal and astrocytic autophagy in the penumbra. *Biomed. Pharmacother.* 90 69–76. 10.1016/j.biopha.2017.03.039 28343073

[B102] Petiot A. Ogier-Denis E. Blommaart E. F. Meijer A. J. Codogno P. (2000). Distinct classes of phosphatidylinositol 3’-kinases are involved in signaling pathways that control macroautophagy in HT-29 cells. *J. Biol. Chem.* 14 992–998. 10.1074/jbc.275.2.992 10625637

[B103] Puyal J. Vaslin A. Mottier V. Clarke P. G. (2009). Postischemic treatment of neonatal cerebral ischemia should target autophagy. *Ann. Neurol.* 66 378–389. 10.1002/ana.21714 19551849

[B104] Qi X. Okuma Y. Hosoi T. Nomura Y. (2004). Edaravone protects against hypoxia/ischemia-induced endoplasmic reticulum dysfunction. *J. Pharmacol. Exp. Ther.* 311 388–393. 10.1124/jpet.104.069088 15178695

[B105] Qin A. P. Liu C. F. Qin Y. Y. Hong L. Z. Xu M. Yang L. (2010). Autophagy was activated in injured astrocytes and mildly reased cell survival following glucose and oxygen deprivation and focal cerebral ischemia. *Autophagy* 6 738–753. 10.4161/auto.6.6.12573 20574158

[B106] Rami A. Langhagen A. Steiger S. (2008). Focal cerebral ischemia induces upregulation of Beclin 1 and autophagy-like cell death. *Neurobiol. Dis.* 29 132–141. 10.1016/j.nbd.2007.08.005 17936001

[B107] Rissanen A. Sivenius J. Jolkkonen J. (2006). Prolonged bihemispheric alterations in unfolded protein response related gene expression after experimental stroke. *Brain Res.* 4 60–66. 10.1016/j.brainres.2006.02.095 16684512

[B108] Roussel B. D. Kruppa A. J. Miranda E. Crowther D. C. Lomas D. A. Marciniak S. J. (2013). Endoplasmic reticulum dysfunction in neurological disease. *Lancet Neurol.* 12 105–118. 10.1016/s1474-4422(12)70238-7 23237905

[B109] Rubinsztein D. C. Codogno P. Levine B. (2012). Autophagy modulation as a potential therapeutic target for diverse diseases. *Nat. Rev. Drug Discov.* 11 709–730. 10.1038/nrd3802 22935804 PMC3518431

[B110] Russell R. C. Tian Y. Yuan H. Park H. W. Chang Y. Y. Kim J. (2013). ULK1 induces autophagy by phosphorylating Beclin-1 and activating VPS34 lipid kinase. *Nat. Cell Biol.* 15 741–750. 10.1038/ncb2757 23685627 PMC3885611

[B111] Ryan F. Khodagholi F. Dargahi L. Minai-Tehrani D. Ahmadiani A. (2018). Temporal pattern and crosstalk of necroptosis markers with autophagy and apoptosis associated proteins in ischemic hippocampus. *Neurotox Res.* 34 79–92. 10.1007/s12640-017-9861-3 29313217

[B112] Rzymski T. Milani M. Pike L. Buffa F. Mellor H. R. Winchester L. (2010). Regulation of autophagy by ATF4 in response to severe hypoxia. *Oncogene* 5 4424–4435. 10.1038/onc.2010.191 20514020

[B113] Schneeloch E. Wenkel S. Mies G. Paschen W. (2004). Spreading depression activates unfolded protein response. *Neurosci. Lett.* 16 37–40. 10.1016/j.neulet.2004.06.061 15342130

[B114] Sheng R. Liu X. Q. Zhang L. S. Gao B. Han R. Wu Y. Q. (2012). Autophagy regulates endoplasmic reticulum stress in ischemic preconditioning. *Autophagy* 8 310–325. 10.4161/auto.18673 22361585

[B115] Sheng R. Zhang L. S. Han R. Liu X. Q. Gao B. Qin Z. H. (2010). Autophagy activation is associated with neuroprotection in a rat model of focal cerebral ischemic preconditioning. *Autophagy* 6 482–494. 10.4161/auto.6.4.11737 20400854

[B116] Shi R. Weng J. Zhao L. Li X. M. Gao T. M. Kong J. (2012). Excessive autophagy contributes to neuron death in cerebral ischemia. *CNS Neurosci. Thera.* 18 250–260. 10.1111/j.1755-5949.2012.00295.x PMC6493486 22449108

[B117] Sokka A. L. Putkonen N. Mudo G. Pryazhnikov E. Reijonen S. Khiroug L. (2007). Endoplasmic reticulum stress inhibition protects against excitotoxic neuronal injury in the rat brain. *J. Neurosci.* 24 901–908. 10.1523/jneurosci.4289-06.2007 PMC6672923 17251432

[B118] Szydlowska K. Tymianski M. (2010). Calcium, ischemia and excitotoxicity. *Cell Calcium* 47 122–129. 10.1016/j.ceca.2010.01.003 20167368

[B119] Tajiri S. Oyadomari S. Yano S. Morioka M. Gotoh T. Hamada J. I. (2004). Ischemia-induced neuronal cell death is mediated by the endoplasmic reticulum stress pathway involving CHOP. *Cell Death Differ.* 11 403–415. 10.1038/sj.cdd.4401365 14752508

[B120] Thiebaut A. M. Gauberti M. Ali C. Martinez De Lizarrondo S. Vivien D. Yepes M. (2018). The role of plasminogen activators in stroke treatment: fibrinolysis and beyond. *Lancet Neurol.* 17 1121–1132. 10.1016/S1474-4422(18)30323-5 30507392

[B121] Tian F. Deguchi K. Yamashita T. Ohta Y. Morimoto N. Shang J. (2010). In vivo imaging of autophagy in a mouse stroke model. *Autophagy* 6 1107–1114. 10.4161/auto.6.8.13427 20930570

[B122] Uchiyama Y. (2001). Autophagic cell death and its execution by lysosomal cathepsins. *Arch. Histol. Cytol.* 64 233–246. 10.1679/aohc.64.233 11575420

[B123] Urbanek T. Kuczmik W. Basta-Kaim A. Gabryel B. (2014). Rapamycin induces of protective autophagy in vascular endothelial cells exposed to oxygen-glucose deprivation. *Brain Res.* 17 1–11. 10.1016/j.brainres.2014.01.017 24462935

[B124] Wang J. Y. Xia Q. Chu K. T. Pan J. Sun L. N. Zeng B. (2011). Severe global cerebral ischemia-induced programmed necrosis of hippocampal CA1 neurons in rat is prevented by 3-methyladenine: a widely used inhibitor of autophagy. *J. Neuropathol. Exp. Neurol.* 70 314–322. 10.1097/NEN.0b013e31821352bd 21412169

[B125] Wang N. Yang L. Zhang H. Lu X. Wang J. Cao Y. (2018). MicroRNA-9a-5p alleviates ischemia injury after focal cerebral ischemia of the rat by targeting ATG5-mediated autophagy. *Cell. Physiol. Biochem.* 45 78–87. 10.1159/000486224 29316542

[B126] Wang P. Guan Y. F. Du H. Zhai Q. W. Su D. F. Miao C. Y. (2012). Induction of autophagy contributes to the neuroprotection of nicotinamide phosphoribosyltransferase in cerebral ischemia. *Autophagy* 8 77–87. 10.4161/auto.8.1.18274 22113203

[B127] Wang P. Xu T. Y. Wei K. Guan Y. F. Wang X. Xu H. (2014). ARRB1/beta-arrestin-1 mediates neuroprotection through coordination of BECN1-dependent autophagy in cerebral ischemia. *Autophagy* 10 1535–1548. 10.4161/auto.29203 24988431 PMC4206533

[B128] Wang Z. Yang W. (2018). Impaired capacity to restore proteostasis in the aged brain after ischemia: implications for translational brain ischemia research. *Neurochem. Int.* 10.1016/j.neuint.2018.12.018 [Epub ahead of print]. PMC6579706 30599146

[B129] Wei H. Perry D. C. (1996). Dantrolene is cytoprotective in two models of neuronal cell death. *J. Neurochem.* 67 2390–2398. 10.1046/j.1471-4159.1996.67062390.x 8931471

[B130] Wei Y. Pattingre S. Sinha S. Bassik M. Levine B. (2008). JNK1-mediated phosphorylation of Bcl-2 regulates starvation-induced autophagy. *Mol. Cell* 20 678–688. 10.1016/j.molcel.2008.06.001 PMC2478643 18570871

[B131] Weidberg H. Shvets E. Shpilka T. Shimron F. Shinder V. Elazar Z. (2010). LC3 and GATE-16/GABARAP subfamilies are both essential yet act differently in autophagosome biogenesis. *EMBO J.* 2 1792–1802. 10.1038/emboj.2010.74 PMC2885923 20418806

[B132] Wen Y. D. Sheng R. Zhang L. S. Han R. Zhang X. Zhang X. D. (2008). Neuronal injury in rat model of permanent focal cerebral ischemia is associated with activation of autophagic and lysosomal pathways. *Autophagy* 4 762–769. 10.4161/auto.6412 18567942

[B133] Xie C. Ginet V. Sun Y. Koike M. Zhou K. Li T. (2016). Neuroprotection by selective neuronal deletion of Atg7 in neonatal brain injury. *Autophagy* 12 410–423. 10.1080/15548627.2015.1132134 26727396 PMC4835980

[B134] Xin X. Y. Pan J. Wang X. Q. Ma J. F. Ding J. Q. Yang G. Y. (2011). 2-methoxyestradiol attenuates autophagy activation after global ischemia. *Can. J. Neurol. Sci.* 38 631–638. 10.1017/s031716710001218x 21672704

[B135] Xing H. Azimi-Zonooz A. Shuttleworth C. W. Connor J. A. (2004). Caffeine releasable stores of Ca2+ show depletion prior to the final steps in delayed CA1 neuronal death. *J. Neurophysiol.* 92 2960–2967. 10.1152/jn.00015.2004 15201305

[B136] Xing S. Zhang Y. Li J. Zhang J. Li Y. Dang C. (2012). Beclin 1 knockdown inhibits autophagic activation and prevents the secondary neurodegenerative damage in the ipsilateral thalamus following focal cerebral infarction. *Autophagy* 8 63–76. 10.4161/auto.8.1.18217 22108007

[B137] Yamamoto H. Kakuta S. Watanabe T. M. Kitamura A. Sekito T. Kondo-Kakuta C. (2012). Atg9 vesicles are an important membrane source during early steps of autophagosome formation. *J. Cell Biol.* 23 219–233. 10.1083/jcb.201202061 PMC3410421 22826123

[B138] Yang Z. Zhao T. Z. Zou Y. J. Zhang J. H. Feng H. (2014). Hypoxia Induces autophagic cell death through hypoxia-inducible factor 1alpha in microglia. *PLoS One* 9:e96509 10.1371/journal.pone.0096509 PMC4018331 24818601

[B139] Yepes M. Roussel B. D. Ali C. Vivien D. (2009). Tissue-type plasminogen activator in the ischemic brain: more than a thrombolytic. *Trends Neurosci.* 32 48–55. 10.1016/j.tins.2008.09.006 18963068

[B140] Yoshida H. Matsui T. Hosokawa N. Kaufman R. J. Nagata K. Mori K. (2003). A time-dependent phase shift in the mammalian unfolded protein response. *Dev. Cell* 4 265–271. 10.1016/s1534-5807(03)00022-4 12586069

[B141] Young A. R. Ali C. Duretete A. Vivien D. (2007). Neuroprotection and stroke: time for a compromise. *J. Neurochem.* 103 1302–1309. 10.1111/j.1471-4159.2007.04866.x 17727635

[B142] Zalckvar E. Berissi H. Mizrachy L. Idelchuk Y. Koren I. Eisenstein M. (2009). DAP-kinase-mediated phosphorylation on the BH3 domain of beclin 1 promotes dissociation of beclin 1 from Bcl-XL and induction of autophagy. *EMBO Rep.* 10 285–292. 10.1038/embor.2008.246 19180116 PMC2658558

[B143] Zhang C. Cuervo A. M. (2008). Restoration of chaperone-mediated autophagy in aging liver improves cellular maintenance and hepatic function. *Nat. Med.* 14 959–965. 10.1038/nm.1851 18690243 PMC2722716

[B144] Zhang T. Liu X. Li Q. Wang J. Jia W. Sun X. (2010). Exacerbation of ischemia-induced amyloid-beta generation by diabetes is associated with autophagy activation in mice brain. *Neurosci. Lett.* 2 215–220. 10.1016/j.neulet.2010.05.064 20553803

[B145] Zhang T. Lu D. Yang W. Shi C. Zang J. Shen L. (2018). HMG-CoA Reductase Inhibitors Relieve Endoplasmic Reticulum Stress by Autophagy Inhibition in Rats With Permanent Brain Ischemia. *Front. Neurosci.* 12:405 10.3389/fnins.2018.00405 PMC6018104 29970982

[B146] Zhang X. Yan H. Yuan Y. Gao J. Shen Z. Cheng Y. (2013). Cerebral ischemia-reperfusion-induced autophagy protects against neuronal injury by mitochondrial clearance. *Autophagy* 9 1321–1333. 10.4161/auto.25132 23800795

[B147] Zhang X. Yuan Y. Jiang L. Zhang J. Gao J. Shen Z. (2014). Endoplasmic reticulum stress induced by tunicamycin and thapsigargin protects against transient ischemic brain injury: involvement of PARK2-dependent mitophagy. *Autophagy* 1 1801–1813. 10.4161/auto.32136 PMC4198364 25126734

[B148] Zheng Y. Q. Liu J. X. Li X. Z. Xu L. Xu Y. G. (2009). RNA interference-mediated downregulation of Beclin1 attenuates cerebral ischemic injury in rats. *Acta Pharmacol. Sin.* 30 919–927. 10.1038/aps.2009.79 19574998 PMC4006642

[B149] Zhou Y. Lee J. Reno C. M. Sun C. Park S. W. Chung J. (2011). Regulation of glucose homeostasis through a XBP-1-FoxO1 interaction. *Nat. Med.* 17 356–365. 10.1038/nm.2293 21317886 PMC3897616

[B150] Zundorf G. Reiser G. (2011). Calcium dysregulation and homeostasis of neural calcium in the molecular mechanisms of neurodegenerative diseases provide multiple targets for neuroprotection. *Antioxid. Redox Signal.* 1 1275–1288. 10.1089/ars.2010.3359 PMC3122891 20615073

